# Identification and Characterization of MicroRNAs in Ovary and Testis of Nile Tilapia (*Oreochromis niloticus*) by Using Solexa Sequencing Technology

**DOI:** 10.1371/journal.pone.0086821

**Published:** 2014-01-23

**Authors:** Jun Xiao, Huan Zhong, Yi Zhou, Fan Yu, Yun Gao, Yongju Luo, Zhanyang Tang, Zhongbao Guo, Enyan Guo, Xi Gan, Ming Zhang, Yaping Zhang

**Affiliations:** 1 Guangxi Key Laboratory of Aquatic Genetic Breeding and Healthy Aquaculture, Guangxi Academy of Fisheries Science, Nanning, Guangxi, China; 2 State Key Laboratory for Conservation and Utilization of Subtropical Agro-bioresources, Animal Reproduction Institute, Guangxi University, Nanning, Guangxi, China; 3 Key Laboratory for Genetic Breeding of Aquatic Animals, Aquaculture Biology, Freshwater Fisheries Research Center, Chinese Academy of Fishery Sciences, Wuxi, Jiangsu, China; 4 State Key Laboratory of Genetic Resources and Evolution and Laboratory of Molecular Biology of Domestic Animals, Kunming Institute of Zoology, Kunming, Yunnan, China; The University of Hong Kong, China

## Abstract

MicroRNAs (miRNAs) are endogenous non-coding small RNAs which play important roles in the regulation of gene expression by cleaving or inhibiting the translation of target gene transcripts. Thereinto, some specific miRNAs show regulatory activities in gonad development via translational control. In order to further understand the role of miRNA-mediated posttranscriptional regulation in Nile tilapia (*Oreochromis niloticus*) ovary and testis, two small RNA libraries of Nile tilapia were sequenced by Solexa small RNA deep sequencing methods. A total of 9,731,431 and 8,880,497 raw reads, representing 5,407,800 and 4,396,281 unique sequences were obtained from the sexually mature ovaries and testes, respectively. After comparing the small RNA sequences with the Rfam database, 1,432,210 reads in ovaries and 984,146 reads in testes were matched to the genome sequence of Nile tilapia. Bioinformatic analysis identified 764 mature miRNA, 209 miRNA-5p and 202 miRNA-3p were found in the two libraries, of which 525 known miRNAs are both expressed in the ovary and testis of Nile tilapia. Comparison of expression profiles of the testis, miR-727, miR-129 and miR-29 families were highly expressed in tilapia ovary. Additionally, miR-132, miR-212, miR-33a and miR-135b families, showed significant higher expression in testis compared with that in ovary. Furthermore, the expression patterns of the miRNAs were analyzed in different developmental stages of gonad. The result showed different expression patterns were observed during development of testis and ovary. In addition, the identification and characterization of differentially expressed miRNAs in the ovaries and testis of Nile tilapia provides important information on the role of miRNA in the regulation of the ovarian and testicular development and function. This data will be helpful to facilitate studies on the regulation of miRNAs during teleosts reproduction.

## Introduction

MicroRNAs (miRNA) are small noncoding RNA molecules (18∼22 nucleotides), which post-transcriptionally regulate the endogenous genes expression, shaping the transcriptome and protein production [1], [2], [3]. Since the first miRNA *lin-14* was characterized in *Caenorhabditis elegans* development, thousands of miRNAs were found in animals and plants by genetic methods and sequencing of small RNA libraries [4]. After endoribonuclease processing by Drosha (also known as Rnasen) and Dicer enzymes, pre-miRNA was digested and then obtained miRNA, which regulate gene expression via binding with the targeting specific sites in the 3′-untranslated region (3′-UTR) of mRNAs [5], [6], [7].

Compared to other vertebrate, teleost reproduction presents many original features. As the variety of species and changes of aquatic environment habitated, fish production strategies (including oviparity, viviparity and ovoviviparity) are diversified for long term adaptations [8]. The most common reproductive strategy in marine is oviparity, including 90% of bony and 43% of cartilaginous fish. Among the oviparity fish, two different types were characterized: 1. annual-spawning fish with the oocytes developing synchronously, like the salmonids; 2. multiple-spawning fish with spawning multiple times during the breeding season and asynchronously ovaries develop, such as cyprinids and tilapia. Tilapias are gonochorism with a short spawning cycle (14 days), and the larva offsprings are monosex [9]. Thus, the study on molecular mechanism of gonads in tilapias will aid in information to understand the sex differentiation of teleosts.

The main regulators of gonadal development and differentiation appear to be the steroid hormones in hypothalamic-pituitary-gonad (HPG) axis [10], [11]. Gonadotropin-releasing hormone (GnRH), follicle-stimulating hormone (FSH) and luteinizing hormone (LH) play important role in oocytes maturation and ovulation. In recent studies, more and more evidences present that miRNA expression are associated with the progress of gametogenesis and gonadal development. In mice, Mir-17–92 (Mirc1) cluster and Mir-106b-25 (Mirc3) miRNA cluster possibly functionally cooperate in regulating spermatogonial development [12]. Moreover, genome-wide miRNA expression has been examined in the gonad of mice (*mus musculus*) [13], cattle (*Bos taurus*) [14], pig (*Sus scrofa*) [15] and goat (*Capra hircus*) [16]. Yu et al demonstrated that in mammalian testis, Mirn122a down-regulated transition protein 2 mRNA expression by mRNA cleavage [17]. Furthermore, Mishima et al have found that 49 miRNA were only detected in mouse testis and 48 miRNAs exclusively expressed in mouse ovary [13]. These results suggested that the organ-specific miRNA played a critical role in testis and ovary development stages. Apart the reports in mammals, miRNAs were extensively studied in teleost fish. Recently, using deep RNA sequencing, miRNA transcriptome of rainbow trout (*Oncorhynchus mykiss*) egg were investigated [18]. Juanchich et al revealed that 13 miRNAs were differentially expressed during oogenesis, which provided strong evidence of major player of miRNAs in oogenesis [19]. Using SOLiD sequencing technology, it had been found sexually dimorphic expression of several miRNAs were abundant in gonads in Atlantic Halibut (*Hippoglossus hippoglossus*) [20].

Nile tilapia (*Oreochromis niloticus*) is an extensively cultured fish species in aquaculture. Because the growth rate of male Nile tilapia is significantly higher than that of females during grow-out period, it is necessary to investigate the molecular mechanism of sex determination and maintenance. Several studies have been carried out for the reproduction and physiology of Nile tilapia. The sex determining genes were isolated and analyzed the expression profile and function in Nile tilapia gonads, such as Cyp19a, Foxl2, Amh and Sox9a,b [21]. As the steroidogenic enzymes responsible for the synthesis of estrogens hormone in fish, the P450 aromatases activity and expression were significantly increased during process of vitellogenesis [22]. DMRT and Amh were exclusively expressed in testis and ovary, which indicated their function in gonadal development [23]. Moreover, Tao and his colleague used RNA-Seq technology to sequence the transcriptomes of XX and XY gonads at four developmental stages [24]. The results revealed that 69 and 259 genes were specifically expressed in XX and XY gonads, respectively [24]. Although some previous reports have provided the expression profiles of several key steroidogenic enzyme genes and the transcriptomic levels in early developmental stages of tilapia, the exact roles of miRNA during tilapia sex determination and differentiation are still unknown. For this reason, further identification of the expression patterns of miRNA involved in gonad differentiation will help us to reveal the network regulation of sex determination in Nile tilapia. In the present study, the aim was to identify the miRNA expressed in the testis and ovary of Nile tilapia. These data will provide new information on the role of miRNA in gonad function and facilitate studies on the reproduction of tilapia.

## Materials and Methods

### Ethics Statement

All the fish were anesthetized with 2-phenoxyenthanol before being euthanized. The fish were cared for in accordance with the Regulations for the Administration of Affairs Concerning Experimental Animals for the Science and Technology Bureau of China throughout the study.

### Collection of Ovaries and Testes Tissues

For Solexa Sequencing, eight healthy adult Nile tilapias (*Oreochromis niloticus*), four males and four females, were provided by Guangxi Academy of Fishery Sciences. After anaesthetized with 2-phenoxyethanol (Sigma-Aldrich, St Louis, MO, USA), the adult males and females were executed and the gonads were excised. Then the tissues were immediately frozen by liquid nitrogen and stored at −80°C until RNA isolation. The total RNA was extracted by Trizol reagent (Invitrogen, Carlsbad, CA, USA) according to the manufacturer’s protocol. After isolation, the quantity was assayed by using TBS 380 Picogreen (Invitrogen, Carlsbad, CA, USA).

For different stage-specific expression, the fishes were anaesthetized with 2-phenoxy-ethanol (Sigma, St. Louis, MO, USA) added to water at the sampling time points [30 days post fertilization (DPF), testis n = 5, ovary n = 5; 50 DPF testis n = 5, ovary n = 5; 75 DPF testis n = 5, ovary n = 5; 100 DPF testis n = 5, ovary n = 5; 165 DPF testis n = 5, ovary n = 5]. After excised the testes and ovaries, the expression levels were assayed by quantitative realtime PCR.

### Small RNA Library Construction and Small RNA Deep Sequencing

For each sample, 1 µg total RNA was used for small RNA direct sequencing analysis by Hiseq2000 in Illumina Genome Analyzer (Illumina, San Diego, CA, USA). After small RNAs of 16–32 nt in length were isolated from the total RNA by size fractionation in a 15% TBE urea polyacrylamide gel, small RNAs were ligated to a 3′ adaptor and 5′ adaptor. Using the RT primer, the products were reverse transcribed to create cDNA constructs. Subsequently, PCR reaction was performed using primers complementary to the two adaptors. Following the purification of the amplified cDNA constructs, the products were sequenced by using Hiseq2000 technology. All sequencing reads were deposited in the Short Read Archive (SRA) database (http://www.ncbi.nlm.nih.gov/sra/), which are retrievable under the accession number SRA115473.

### Bioinformatic Analysis

After adaptor trimming, removal of low-quality sequences and orphan reads, reads of 18–32 nt in length were kept for further bioinformatics analysis. Then the remaining reads were mapped to Nile tilapia genome with a tolerance of one mismatch in the seed sequence. Subsequently, by BLAST against the Rfam database (11.0, http://Rfam.sanger.ac.uk/), the reads mapped to the Nile tilapia genome were analyzed to discarded the rRNA, tRNA, snRNA, ncRNA and other snoRNA sequences from the small sequences. After that, the remaining sequences were identified for the conserved miRNAs in Nile tilapia by a BLAST search against the miRNA database, miRBase (version18.0, http://www.mirbase.org/). To discuss the differential miRNA in testis and ovaries in Nile tilapia, miRNA was normalized to obtain the expression of transcripts using the DEGseq software (http://www.bioconductor.org/packages/release/bioc/html/DEGseq.html). To understand the function of the miRNA found in testis and ovaries, potential target sequences for the newly identified miRNA were predicted by integrating three databases (TargetScan, PicTar and miRanda). Furthermore, Gene ontology (GO) annotation and KEGG pathway analysis were performed to identify the functional modules regulated by the miRNA.

### Quantitative Realtime PCR

To validate and characterize the differentially expressed miRNAs identified in testis and ovaries of Nile tilapia, miRNAs were selected and analyzed their relative expression by quantifying the miRNA stem-loop. The total RNAs were isolated using RNA isolation kit (Bio-Rad, Richmond, CA), following cDNA generated using 1 µg of total RNA by reverse transcription kit (Invitrogen, Carlsbad, CA). Quantitative realtime PCR was performed on ABI 7500 PCR system (Applied Biosystems, Foster City, CA) and using qPCR/Real-Time PCR Reagents provided by Bio-rad (Richmond, CA). The primers were showed in supplement data (Table S1 in File S1). The PCR reactions were performed as follows: 10 minutes at 95°C, and then 40 cycles of 30 seconds at 95°C, 1 minute at 60°C. For each sample, the samples were tested in triplicate. Finally, polyacrylamide gel electrophoresis and melting curve were performed to confirm if the amplifications were specific. β-actin was used as an internal control.

### Statistical Analysis

The data of quantitative Realtime PCR were expressed as means ± SD and the significant differences were confirmed by Turkey’s multiple range tests using SPSS 13.0 software. P values <0.05 were taken to indicate statistical significance.

## Results

### General Features of Small RNAs in Gonads of Tilapia

To investigate miRNAs differentiation between ovary and testis, two small RNA libraries were generated from 150 DPF tilapia ovaries and testis, respectively. Subsequently, the two libraries were following a pipeline of Illumina small RNA deep sequencing. In total, 9,731,431 and 8,880,497 raw reads were obtained from the sexually mature ovaries and testes, respectively. After filtering the low-quality sequences, empty adaptors and single-read sequences, 8,061,686 and 7,502,205 clean reads of 18–32 nt were selected further analysis from libraries of the ovaries and testes, respectively. Among the clean reads, 5,407,800 sequences from the ovaries and 4,396,281 sequences from the testes mapped perfectly to the tilapia genome (Table S2 in File S1), amounting to 67.08% and 58.60% of the total reads, respectively. Among them, 1,432,210 reads in ovaries and 984,146 reads in testes were matched with miRNAs in Rfam database. The rest of the read were matched with other types of RNA, including tRNA, rRNA, snRNA or snoRNA. The ratios and counts of the small RNAs type were showed in Table S2 in File S1.

**Figure 1:**
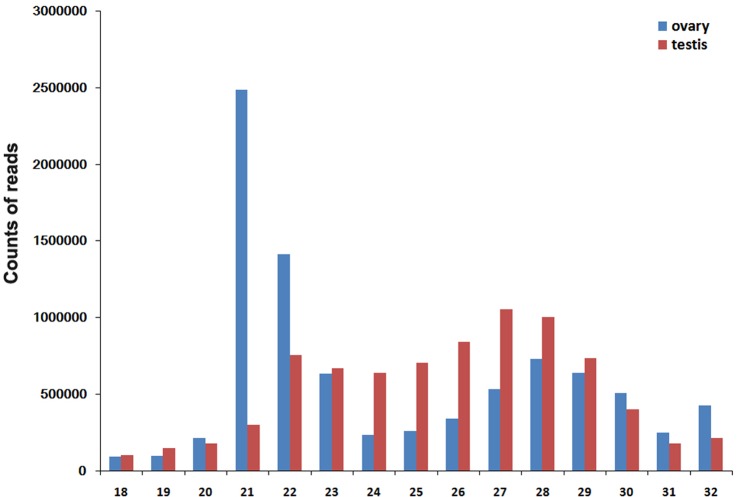
Length distribution of small RNAs sequences in ovary and testis of Nile tilapia. Sequence length distribution of clean reads based on the abundance; the most abundant size class of ovary was 21 nt, followed by 22 nt and 23 nt while the most abundant size class of testis was 27 nt, followed by 28 nt and 26 nt.

The length distribution of the sequenced small RNAs (sRNAs) was difference between ovaries and testes. The most abundant length class in ovaries was 21 amounting to 28.14%, while the most abundant size class in testes was 27 nt amounting to 13.33%. Moreover, the length distribution of the miRNAs was more even in testes than ovaries (Figure 1).

### Identification of Known miRNAs in Tilapia

To investigate the expression of known miRNAs in the ovaries and testes, small RNA sequences identified by Illumina small RNA deep sequencing were compared with known mature miRNAs and precursors in miRBase (miRBase18). The results showed that 764 mature miRNA, 209 miRNA-5p and 202 miRNA-3p were found in the two libraries, and 525 known miRNAs are both expressed in the two libraries (Table S3 in File S1).

The Illumina small RNA deep sequencing approach allows us to determine the relative abundance of various miRNA families by calculating the sequencing frequency. A highly expressed miRNA will likely have a large number of sequenced clones. Between the ovaries and testes libraries, known miRNAs showed a broad range of expression levels: several miRNAs (such as miR-181a and miR-143) were abundant expressed more than hundreds of thousands of sequence reads, while some (such as miR-4832 and miR-92) miRNAs showed less than 10 reads (Table S3 in File S1), which suggests that the expression levels were very different. The proportion of different categories of small RNAs often reflects the roles in a particular tissue or different developmental stages and their associated biological mechanisms. The following 4 miRNAs were dominantly expressed in the two libraries: miR-181a, miR-181a-5p, miR-143 and miR-143-3p. Each of these miRNAs had more than 100,000 reads either in ovaries and testes, while the majority of other miRNAs sequenced had more than 500 reads. The most abundant miRNA in the ovaries were miR-181a and miR-181a-5p, which both had 297,698 reads. However, in the testes, the highest expressed miRNA was miR-143 and miR-143-3p, which both was sequenced for 172,512 reads (Table S3 in File S1).

Sequence analysis indicated that the relative abundance of members within one miRNA family varied greatly in the tilapia, suggesting functional divergence within the family. For example, in the ovaries and testes, miR-30 family abundance varied from 1 reads and 1 reads (miR-30f) to 10144 and 10383 (miR-30e-5p), respectively. These results indicated that different members within one miRNA family can have clearly different expression levels, most likely due to tissue- or developmental stage-specific expression.

### Multiple isomiRs in Tilapia

**Figure 2:**
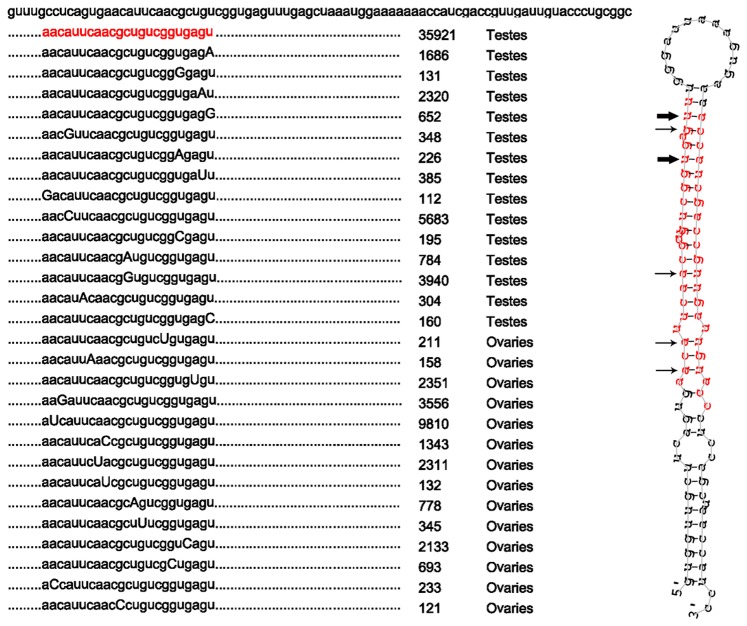
Details of miR-181-a isomiRs including sequence counts. The secondary structure miRNA precursor and counts of multiple isomiRs are presented. Single nucleotide substitutions are highlighted by capital letters. The reference miRNA sequence is shown in red. The dominant cleavage sites are showed by arrows. Bold arrows show the most abundant isomer cleavage sites, whereas small arrows indicate secondary abundance isomiRs cleavage sites.

The recent studies suggested that isomiRs were observed using miRNA deep sequencing which is encoded by the same sequences from genome but show variations from the reference miRNAs [25], [26]. The present study indicated that multiple isomiRs was found and we illustrated the most abundant miRNA (miR-181-a) in gonads as example. In total, 29 isomiRs (the counts are more than 100) were confirmed as miR-181-a by compared with Rfam database. Among all the single nucleotide substitutions, the most dominate substitution is A-U (3.45%). Meanwhile, the result suggested that the most variation sites are U19 and U23 with three different mutant genotypes. This is corresponding with other researches that the most abundant variation sites showed a 3′-bias. The two most variation sites are at the end of 3′ domain, while the sites with two different mutant genotypes could be found in the 5′ and central domain. Nevertheless, the isomiRs genotype showed that the 3′ end is more variable than other areas (Figure 2).

### Differential Expression of miRNAs in Ovaries and Testes

**Figure 3:**
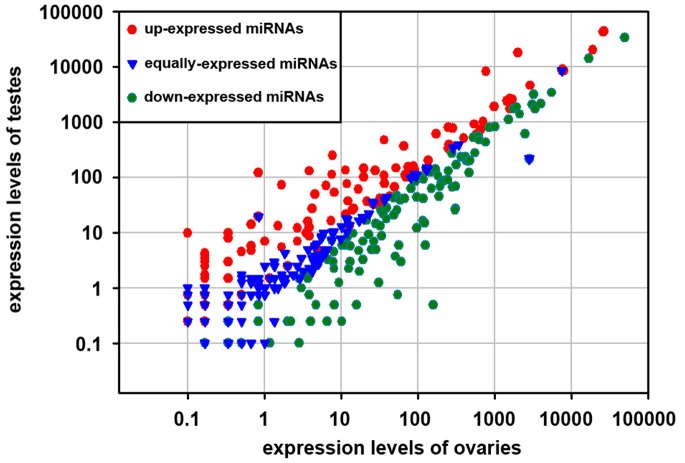
Comparison of expression levels of miRNAs in ovary and testis. The X and Y axes show expression levels of miRNAs in ovary and testis, respectively. Red dots represent up-expressed miRNAs; green dots represent down-expressed miRNAs; blue dots represent equally-expressed miRNAs; the expression level in ovary was regareded as the control.

The major objective of the present study is to illustrate the differential expression between mature ovaries and testes in tilapia. Based on the deep sequencing results, the miRNA expression levels could be calculated relatively (Table S4 in File S1). Compared with testes, 246 miRNAs were significantly up-regulated (p-value <0.01) while 184 miRNAs were down-regulated (p-value <0.01) in ovaries (Table S4 in File S1, Figure 3). Among the up-regulated miRNAs, miR-727-3p showed a greater expression than 300-fold in ovaries compared with that in testes, followed by miR-129b-3p, miR-129-2-3p, miR-129a-3p, miR-129-1-3p, miR-29d, miR-29-3p and miR-727-5p with more than 30-fold. In contrast, the most down-regulated miRNAs in ovaries compared with that in testes is miR-132a which showed a greater expression levels than 147-fold, followed by miR-132-5p, miR-212, miR-132, miR-132-3p, miR-132b, miR-33a, miR-212-5p and miR-135b with more than 30-fold.

**Figure 4:**
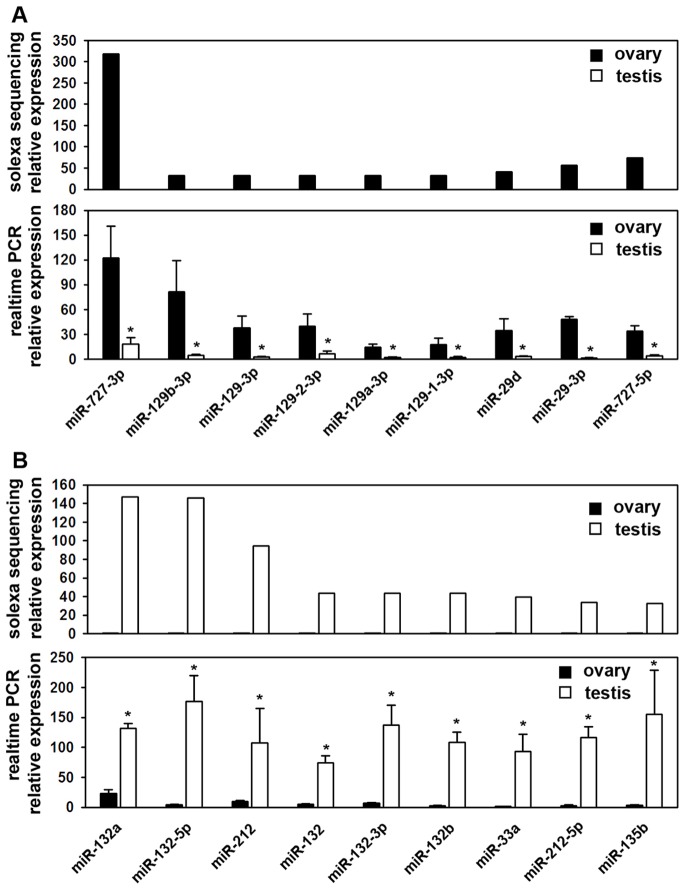
Realtime-PCR validation of differentially expressed miRNAs inidentified using solexa small RNA deep sequencing. A. Fold changes of nine miRNAs that were up-regulated differentially expressed in ovary and testis using deep sequencing data and realtime-PCR. * indicate significant difference (P<0.05). B. Fold changes of nine miRNAs that were down-regulated differentially expressed in ovary and testis using deep sequencing data and realtime-PCR. * indicate significant difference (P<0.05).

To validate the differential expression results based on Illumina small RNA sequence technology, the most different expression of six miRNA transcripts (miR-727-3p, miR-727-5p, miR-29-3p, miR-132a, miR-132-5p and miR-212) were assayed by quantitative realtime PCR (Figure 4). The results were similar with the Illumina small RNA sequence data. These results showed that the approach using small RNA deep sequencing is a reliable method for identifying differential expression in gonads of Nile tilapia.

### Developmental Expression Patterns of miRNAs in the Gonads

To further characterize the functionality of these differentially expressed miRNAs identified from the tilapia gonads, the expression levels of miR-727-3p, miR-129b-3p, miR-129-3p, miR-129-2-3p, miR-129a-3p, miR-132a, miR-132-5p, miR-212, miR-132 and miR-132-3p were further examined in gonads from 30, 50, 75, 100, and 165 DPF tilapia in both males and females (n = 5).

**Figure 5:**
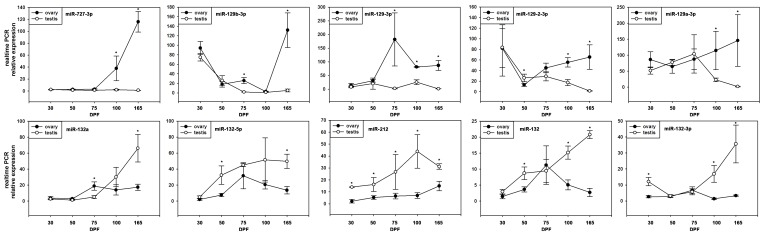
Expression patterns of miR-727-3p, miR-129b-3p, miR-129-3p, miR-129-2-3p, miR-129a-3p, miR-132a, miR-132-5p, miR-212, miR-132 and miR-132-3p in different developmental stages of tilapia gonads were assayed by realtime-PCR. * indicate significant difference between ovary and testis (P<0.05).

The results showed different stage-specific expression patterns in gonad development. The expression patterns of miR-727-3p and miR-129-3p are similar; the lower expression was observed among all the development stages in testis while increased dramatically in 100, and 75 DPF in ovary, respectively. However, the expression dynamics of miR-129b-3p and miR-129-2-3p were different; the expression was decreased from 30 DPF in both testis and ovary while increased dramatically to peak in 165 DPF only in ovary. In contrary, miR-129a-3p showed no significant change in ovary while met a significant decreasing in 100 DPF in testis (Figure 5).

For the miRNAs that showed higher expression in adult ovary than that in testis, the stage-specific expression patterns were similar. All the tested miRNAs showed stable low expression in testis while raised generally in all the stages and reached the peak in 165 DPF in ovary (Figure 5).

### Target Prediction and Function Annotation

**Figure 6:**
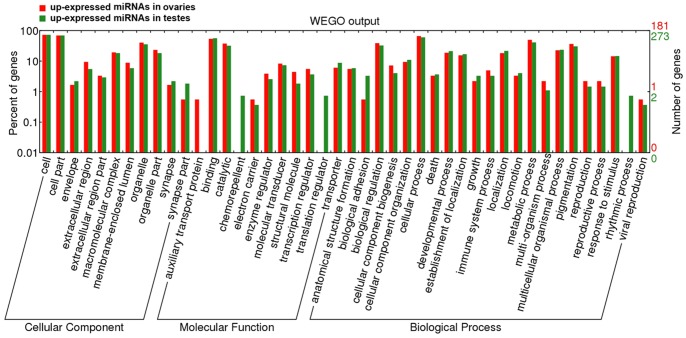
Gene ontology (GO) classification annotated for predicted target genes of 454 differentially expressed miRNAs. The figure shows partial GO enrichment for the predicted target genes in molecular function, cellular component and biological processes.

To further illustrate the biological processes and physiological functions of the differentially expressed miRNAs between tilapia ovaries and testes, the target genes was predicted by integrating TargetScan, PicTar and miRanda databases. In total, 9486 target genes were found (data is not shown). Further, the Gene ontology (GO) annotation and KEGG pathway analysis were carried out to identify functional genes regulated by the differential expression miRNAs (Figure 6). As for molecular function level, the up-expressed miRNAs in ovaries target genes showed significant higher counts in auxiliary transport protein GO term while the target genes of up-expressed miRNAs in ovaries had higher counts in chemorepellent and translation regulator GO terms. As for molecular function level, biological adhesion and rhythmic process showed more counts in target genes of up-expressed miRNAs in testes. The KEGG pathway analysis suggested 222 pathways showed with the target genes of differential expression miRNAs. The results indicated that tryptophan metabolism, nucleotide excision repair, galactose metabolism, fatty acid degradation, steroid hormone biosynthesis, geraniol degradation and pathways in cancer showed higher counts in the target genes of up-expressed miRNAs in ovaries. In contrast, target genes of Calcium signaling pathway, HTLV-I infection, proteoglycans in cancer, metabolic pathways and neuroactive ligand-receptor interaction had higher counts which showed more active of these miRNAs participated in these pathways (Table 1).

**Table 1:** The significant difference pathways of target genes between mature ovaries and testes.

Pathways ID	Pathways	Gene counts for ovaries	Gene counts for testes	P-value
ko00380	Tryptophan metabolism	57	3	0.0038
ko03420	Nucleotide excision repair	53	7	0.0013
ko00052	Galactose metabolism	21	1	0.0010
ko00071	Fatty acid degradation	11	4	0.0320
ko00140	Steroid hormone biosynthesis	36	11	0.0243
ko00281	Geraniol degradation	47	21	0.0083
ko05200	Pathways in cancer	25	8	0.0115
ko04020	Calcium signaling pathway	47	7	0.0216
ko05166	HTLV-I infection	4	49	0.0001
ko05205	Proteoglycans in cancer	41	117	0.0427
ko01100	Metabolic pathways	6	41	0.0321
ko04080	Neuroactive ligand-receptor interaction	2	41	0.0243

## Discussion

In this study, we reported a comprehensive annotation and analysis of Nile tilapia miRNA expressed in ovary and testes. The length analysis of sequences presented that the dominant size of small RNAs in ovary was 21 nt followed by 22 and 23 nt sequences, while in testis, 27 nt size is the predominant size followed by 28 nt and 26 nt. This result shows a duality of length distribution of small RNAs in tilapia testis and ovary. In mouse ovary, the most abundant small RNA length has been confirmed between 21–23 nt [27]. However, the dominated small RNA length in mouse testis is 27 nt [28]. This is due to the abundant expression of piRNAs in mammalian testis. The novel class of small RNA piRNA was found in 2006 which shows a length distribution as 25–30 nt [29], [30], [31]. In contrast with mammalians, both ovary and testis showed a significant piRNA expression in zebrafish [32]. Thus, the length distribution of small RNAs in zebrafish is dominated as 28 nt length [33]. In *Xenopus laevis*, the piRNAs also showed expression in both female and male gonads [34]. These clues suggest that similar constitution of small RNAs in low vertebrates. In the present study, the tilapia is similar result with mammalians which showed a duality between ovary and testis. Up to date, the data of small RNA in cultured teleosts including tilapia are still limited, especially lack of the information about small RNAs family in gonads. Thus, the constitution of small RNAs is still blurry in tilapia. In a word, we showed the phenomenon that different length distribution in tilapia ovary and testis while the reason needs to be unveiled in further study.

In the mature tilapia gonads, the most abundant sequenced miRNAs were miR-181a, miR-181a-5p, miR-143 and miR-143-3p which expressed more than hundreds of thousands of sequence reads. The miR-181a family also expresses abundantly in primordial germ cells (PGCs) of the chicken [35], as well as in both ovary and testis in human [36] and mouse [37]. Zhou and colleagues reported that sirtuin-1 is the target of miR-181a [38]. Sirtuin-1 null mice showed to be sterile in males and sirtuin-1 also participated with growth hormone/insulin-like growth axis [39]. These clues strongly supported that miR-181a family play a curial role in reproductive. Similar with the present result, miR-143 has been reported highly expressed in gonads of human [36], goat [16] and Atlantic halibut [20]. Several studies showed that miR-143 could induce adipose cell differentiation and fat deposition [40]. In addition, miR-143 has been regarded as a regulator in endocrine system in mammalian gonads and pregnancy maintenance [41], [42], [43]. In a word, the abundant expressed miRNAs in tilapia gonad, including ovary and testis are functionally conserved with other vertebrates that these miRNAs may play crucial role in reproductive physiology.

Previous studies have shown that isomiRs are commonly exist in miRNA cloning library, which are generated from the same hairpin pre-miRNA and display variations of sequence from the reference miRNAs in miRbase [25], [26]. In our study, the results that multiple isomiRs were found in testis and ovaries miRNA were consistent with prior reports. The phenomenon of multiple isomiRs was again observed in Channel Catfish (*Ictalurus punctatus*) tissues miRNA library and human embryonic stem cells miRNA library [25], [44]. During the pre-miRNA editing, single nucleotide substitutions including transition and transversition were represent, with 3.45% A-U substitution in our study. Furthermore, the most variation site in Nile tilapia testes and ovaries miRNA are U19 and U23, which are at the end of 3′ domain. The result is corresponding with the researches that the most abundant variation sites are in 3′-bias. These isomiRs featuring slight variation at terminal sequence of annotated matured miRNA were due to the variation site of cleavage by Dicer and thus making a pool of isomiRs [45].

Up to date, although several studies have been reported on miRNAs expression in gonads of higher vertebrates, research of teleosts gonads miRNAs has seldom showed. The present result indicates that miR-727, miR-129 and miR-29 family showed significant increasing expression in ovary compared with that in testis. In limited miRNAs information of teleosts gonads, miR-727 was founded in Zebrafish brain [46]. Also miR-727 expressed in tilapia muscle [47]. However, the functional details of miR-727 have not been illustrated clearly. In contrast with miR-727, the miR-129 and miR-29 family were deeply elucidated, even in gonads development. It was predicted that miR-129 acts as a physiological suppressors for secretory activity. The target genes of miR-129 family are cell differentiation and growth regulators, including CDK4, CDK6, GALNT1 and SOX4 [48]. Dyrskjøt and colleagues demonstrated that miR-129 promotes cell death *in vitro* [48]. Meanwhile, via down-regulation of CDK6, miR-129 arrests cell growth in mouse lung epithelial cells [49]. In tilapia, the high expression of miR-129 in ovary may control the cell growth and differentiation in final process of ovary maturation through down-regulation of the target mRNAs. Correspondingly, miR-29 expression levels showed a progressive increasing throughout oogenesis [34], [36]. A proposed function to account for the miR-29 expression during ovulation periods is controlling of ovarian cell steroidogenesis [50]. Therefore, these abundant expressions in ovary play an important role during final stage of eggs maturation.

In contrast with tilapia ovary, the most abundant expressed miRNAs families are miR-132, miR-212, miR-33a and miR-135b in testis. In mammalians, miR-212/132 as tandem miRNA shows a necessity in development and function of neurons. Meanwhile, miR-212/132 has been connected with mammary gland development. The function of miR-212/132 is down-regulation of matrix metalloproteinase 9 (MMP-9) which is an activator of TGFβ [51]. By suppression CITED-1 expression, TGFβ showed anti-proliferative function in epithelial cells and participates with estrogen receptor pathway in mammary gland. No such information has been showed in testis development of miR-212/132. However, considering connection between miR-212/132 and estrogen receptor pathway [51], these clues suggest that miR-212/132 regulate steroid hormone pathway to participate with testis differentiation. Insulin signaling pathway has been demonstrated is required for the maturation of testis in mice [52]. Further, Dávalos et al reported that miR-33a controlled fatty acid regulation by repressing the insulin receptor substrate 2 [53]. Thus, the high expression of miR-33a in tilapia testis may contribute to the maturation of testis via regulation of insulin signaling pathway. Among the miRNAs specific expressed in testis, miR-135b is specifically enriched in adult testis [54] which suggested a crucial role of miR-135b during spermiogenesis.

We also showed the developmental expression patterns of ten differential miRNAs between ovary and testis. Five different time points of sample were collected according to sex differentiation in tilapia: 30 DPF - initial morphological sex differentiation could be distinguished in the gonads; 50 DPF - in males onset of meiosis, and in females immature stage; 75–100 DPF - testes are in active spermatogenesis while oocytes are still in a previtellogenic stage in ovary; 165 DPF - mature fish [55], [56]. Interestingly, the miRNAs which showed a higher expression in testis exhibited different stage-specific expression patterns during gonad development while the higher expressed miRNAs in ovary showed similar expression patterns during gonad development. Several studies showed that sex-biased miRNA expression in adults or developmental dynamic among the stages [13], [14], [35], [44]. However, the different stage-specific expression was rarely reported, especially in teleosts. Herein, we showed different expression patterns in tilapia gonads of miRNAs which suggested the variety function and regulatory miRNAs during sex differentiation. More details about the function and mechanisms of miRNAs during this process should be unveiled in further study.

Despite the studies of miRNAs expression in gonads, limited reports about sexually dimorphic microRNAs are provided in recent. By deep sequencing of adult mouse testis and ovary, miRNAs from adult mouse ovary and testis have been cloned [13], [57], [58]. However, these studies are focus on discover novel miRNAs in these tissues rather than organ-specific expression. Mishima and colleagues proposed that miRNAs originated from X-chromosome showed a male-biased expression [13]. It has been regarded that these miRNAs are related to spermatogenesis. Andrew and colleagues showed sexually dimorphic expression of miRNAs in chicken ovary and testis [59]. They found that miR-101, miR-31 and miR-202-5p are reasonable for gonadal sexual differentiation. In teleosts, the studies on miRNA in sex differentiation are rare. The present research suggested several miRNAs participated with the finally gonads development and sex differentiation. Meanwhile, by realtime PCR, the miRNA differentiation was validated which indicated deep sequencing is a reliable methods to detect expression variation in tilapia tissues.

### Conclusion

The present study is the initial study on miRNA expression profile of ovary and testis in tilapia. In total, 764 mature miRNA, 209 miRNA-5p and 202 miRNA-3p were identified in tilapia gonads and 430 known miRNA that were differentially expressed in ovary and testis which could exert novel functions in sex differentiation. Some miRNA families, such as miR-727, the miR-129 and miR-29 families expressed highly in tilapia ovary suggested a crucial role in the follicular growth or ovulation mechanism in tilapia. On the other hand, some miRNA families, showed significant higher expression in testis compared with that in ovary, which exert potential function in spermatogenesis. Further researches should be concerned the function of these differential expression miRNA which could aid to understand the mechanism of sex differentiation regulated by miRNAs.

## Supporting Information

File S1 Contains 4 parts: Table S1, Table S2, Table S3 and Table S4. Table S1. The primers used in the study. Table S2. Summary of Illumina small RNA deep sequencing data for small RNAs in ovaries and testis of tilapia. Table S3. The expression patterns of known miRNAs in the tilapia ovaries and testes libraries. Table S4. The significantly differential expressed known miRNAs between ovaries and testes.
(XLS)
